# Sleep spindle and slow wave activity in Bipolar Disorder: preliminary observations from a high–density EEG study

**DOI:** 10.1192/j.eurpsy.2023.1076

**Published:** 2023-07-19

**Authors:** C. Sanguineti, F. L. Donati, M. Sala, C. Carrara, C. Casetta, C. Zangani, A. Mayeli, A. Castelnovo, M. P. Canevini, A. D’Agostino

**Affiliations:** 1 Department of Health Sciences; 2 Department of Pathophysiology and Transplantation, Università degli Studi di Milano; 3 San Carlo Hospital, ASST SS. Paolo e Carlo, Milano, Italy; 4 Warneford Hospital, Department of Psychiatry, University of Oxford, Oxford, United Kingdom; 5 Department of Psychiatry, University of Pittsburgh, Pittsburgh, United States; 6 Sleep Center, Neurocenter of Southern Switzerland, Civic Hospital of Lugano, Lugano, Switzerland; 7 San Paolo Hospital, Epilepsy Center - Sleep Medicine Center, Childhood and Adolescence Neuropsychiatry Unit, ASST SS. Paolo e Carlo; 8 Department of Mental Health and Addiction, ASST Santi Paolo e Carlo, Milano, Italy

## Abstract

**Introduction:**

Recent research on Schizophrenia (SCZ) suggests that reduced sleep spindle and slow wave density could be particularly informative of underlying thalamocortical and cortical synchronization mechanisms and dysfunctions. Although sleep disturbances are also highly prevalent across all stages of Bipolar Disorder (BD), the objective evaluation of sleep macrostructure and microstructural oscillatory activity remains understudied in this population.

**Objectives:**

We aimed to investigate sleep EEG activity in BD, with a focus on sleep architecture, sleep spindles and slow waves.

**Methods:**

We recorded high-density EEG (64–channel BrainAmp, Brain Products GmbH, Germany) during sleep in 18 euthymic patients with BD and 18 age/gender-matched healthy control (HC) subjects. After sleep scoring and EEG artifact rejection, several parameters of sleep spindles (12-16 Hz), including density and amplitude, and slow waves (0.1-4 Hz) were identified for the first cycle of sleep using automated algorithms and compared between groups using non-parametric statistics.

**Results:**

BD subjects showed significantly higher Wake After Sleep Onset and lower Sleep Efficiency (Table 1). Total (12 - 16 Hz), slow (12 - 14 Hz) and fast (14 - 16 Hz) sleep spindle parameters of density (Image 1) and amplitude did not differ significantly between groups. On the other hand, slow wave density was reduced in a large frontal cluster of electrodes in the BD group (Image 2).

**Table 1:** 

	**BD (n = 18)**	**HC (n = 18)**	**Difference** **(p value)**
**WASO (min ± sd)**	140,61 ± 74,23	84,34 ± 59,84	0,017
**Sleep efficiency (% ± sd)**	72,47 ± 14,33	82,43 ± 11,58	0,028

**Image:**

**Figure:**
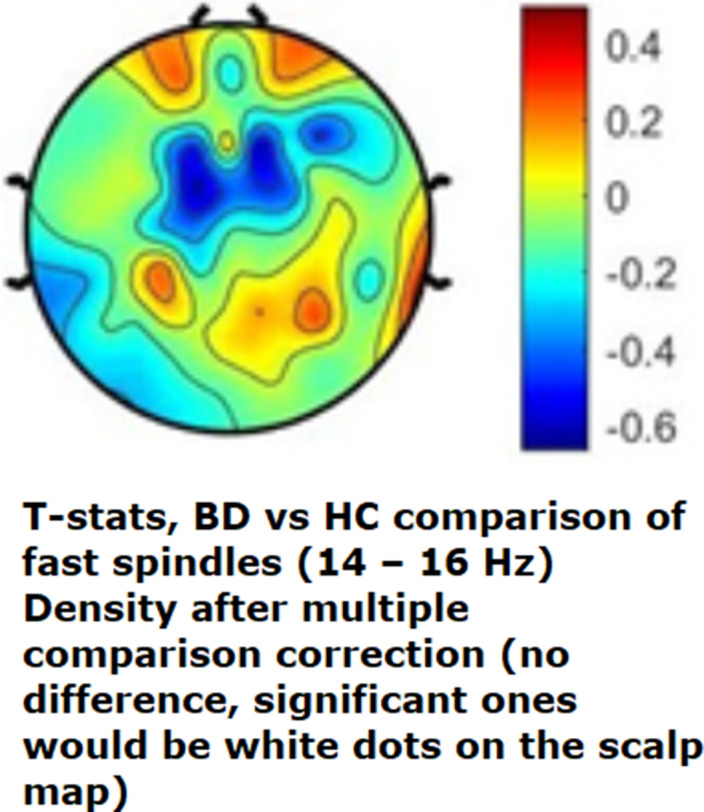

**Image 2:**

**Figure:**
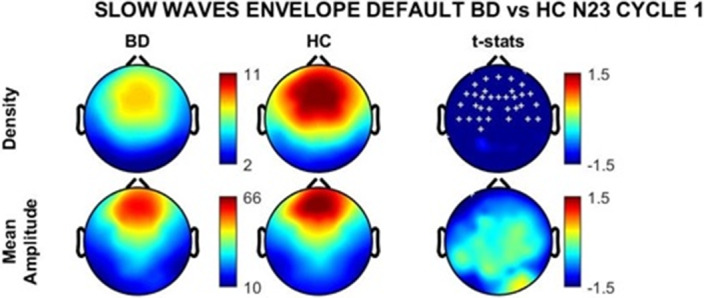

**Conclusions:**

The absence of sleep spindle deficits in the BD group suggests that the systems involved in generating and maintaining these thalamocortical oscillations are presevered during periods of clinical stability in Bipolar Disorder. Conversely, reduced sleep slow wave density points to an altered cortical synchronization, which might represent a common neurophysiological feature shared with Schizophrenia. Further research is needed to confirm these preliminary observations in all–night recordings and with a direct comparison of larger cohorts of patients with both diagnoses.

**Disclosure of Interest:**

None Declared

